# Characteristics of COVID-19 Breakthrough Infections among Vaccinated Individuals and Associated Risk Factors: A Systematic Review

**DOI:** 10.3390/tropicalmed7050081

**Published:** 2022-05-22

**Authors:** Shilpa Gopinath, Angela Ishak, Naveen Dhawan, Sujan Poudel, Prakriti Singh Shrestha, Prabhjeet Singh, Emily Xie, Peggy Tahir, Sima Marzaban, Jack Michel, George Michel

**Affiliations:** 1 Department of Internal Medicine, Division of Infectious Disease, Johns Hopkins University, Baltimore, MD 21205, USA; 2 Division of Research and Academic Affairs, Larkin Community Hospital, South Miami, FL 33143, USA; aishak@larkinhospital.com (A.I.); spoudel@larkinhospital.com (S.P.); prakritisingh@hotmail.com (P.S.S.); smarzban@larkinhospital.com (S.M.); jmichel@larkinhospital.com (J.M.); 3 Department of Bioengineering and Therapeutic Sciences, University of California San Francisco (UCSF), San Francisco, CA 94158, USA; naveen.dhawan@ucsf.edu; 4 Department of Bioengineering, University of California Berkeley, Berkeley, CA 94704, USA; 5 Krieger School of Arts and Sciences, Johns Hopkins University, Baltimore, MD 21218, USA; 6 Bloomberg School of Public Health, Johns Hopkins University, Baltimore, MD 21205, USA; psingh40@jh.edu; 7 Department of Plant and Microbial Biology, University of California, Berkeley, CA 94720, USA; emily.x@berkeley.edu; 8 University of California San Francisco (UCSF) Library, San Francisco, CA 94143, USA; peggy.tahir@ucsf.edu; 9 Department of Internal Medicine, Larkin Community Hospital, South Miami, FL 33143, USA; gmichel@larkinhospital.com

**Keywords:** COVID-19, SARS-CoV-2, variants, reinfections, breakthrough infections, vaccination

## Abstract

We sought to assess breakthrough SARS-CoV-2 infections in vaccinated individuals by variant distribution and to identify the common risk associations. The PubMed, Web of Science, ProQuest, and Embase databases were searched from 2019 to 30 January 2022. The outcome of interest was breakthrough infections (BTIs) in individuals who had completed a primary COVID-19 vaccination series. Thirty-three papers were included in the review. BTIs were more common among variants of concern (VOC) of which Delta accounted for the largest number of BTIs (96%), followed by Alpha (0.94%). In addition, 90% of patients with BTIs recovered, 11.6% were hospitalized with mechanical ventilation, and 0.6% resulted in mortality. BTIs were more common in healthcare workers (HCWs) and immunodeficient individuals with a small percentage found in fully vaccinated healthy individuals. VOC mutations were the primary cause of BTIs. Continued mitigation approaches (e.g., wearing masks and social distancing) are warranted even in fully vaccinated individuals to prevent transmission. Further studies utilizing genomic surveillance and heterologous vaccine regimens to boost the immune response are needed to better understand and control BTIs.

## 1. Introduction

The severe acute respiratory syndrome coronavirus type 2 (SARS-CoV-2) that emerged in December 2019 in Wuhan, China, and was the cause of coronavirus 2019 disease (COVID-19), continues to cause morbidity as part of the ongoing pandemic. As of 4 March 2022, 440,807,756 confirmed cases of COVID-19, including 5,978,096 deaths have been reported [1]. Mortality due to COVID-19 has substantially decreased since the introduction of vaccines and mass vaccination efforts worldwide. As of 27 February 2022, a total of 10,585,766,316 vaccine doses have been administered around the world [1].

However, emerging variants of SARS-CoV-2 and waning immunity in vaccinated individuals continue to hinder efforts to control the disease. Breakthrough infections (BTIs) are defined by the United States (U.S.) Centers for Disease Control and Prevention (CDC) as a positive COVID-19 test by a reverse transcription-polymerase chain reaction (RT-PCR) or rapid antigen test > 14 days following the final dose of the recommended vaccination regimen [2]. In the U.S. as of 22 January 2022, the rate of BTIs was 846.73 per 100,000 in individuals who had completed a primary series of vaccination and 642.19 per 100,000 in those who had completed a primary series and a booster dose [3]. The death rate was 0.96 per 100,000 individuals vaccinated with a primary series and 9.68 in unvaccinated people [3].

The CDC and SARS-CoV-2 Interagency Group (SIG) variant classification define four classes of SARS-CoV-2 variants: variants being monitored (VBM), variants of interest (VOI), variants of concern (VOC), and variants of high consequence (VHC) [4]. VBM includes variants that were associated with an increased rate of transmission but are no longer detected and do not pose a threat to public health in the U.S. These currently include Alpha (B.1.1.7 and Q lineages), Beta (B.1.351, descendent lineages), Gamma (P.1, descendent lineages), Epsilon (B.1.427, B.1.429), Eta (B.1.525), Iota (B.1.526), Kappa (B.1.617.1), 1.617.3, Mu (B.1.621, B.1.621.1), and Zeta (P.2). VOI are associated with increased transmissibility and higher levels of infection. Iota (B.1.526) and B.1.525, identified in the United States, and Zeta (P. 2), first detected in Brazil, belong to this class [4]. Increased transmissibility and disease severity is seen in VOC. These include Alpha (B.1.1.7), first detected in the United Kingdom; Gamma (P.1), first detected in Brazil; Beta (B.1.351) from South Africa; and Epsilon (B.1.427 and B.1.429), detected in the United States [4]. Among all the variants, the Delta (B.1.617.2) variant was reported to have the most transmissibility and severity based on the hospitalization rate until the advent of Omicron (B.1.1.529), which was detected for the first time in South Africa on 24 November 2021 [5,6]. Recent reports suggest that certain VOC might result in a less robust immune response, among other factors, following vaccination against COVID-19, especially in patients with immunosuppression [7,8].

On a mass scale, BTIs pose a serious challenge in tackling the pandemic as these patients may serve as a source of viral spread [9]. With the emergence of VOC such as Omicron and its variants and waning immunity in certain populations after vaccination, a better understanding of BTIs and their attributes, particularly with variant profiles, is essential. These data can help guide public health efforts in determining specific populations that could benefit the most from booster doses of COVID-19 vaccines and help to assess vaccine effectiveness against specific variants.

We explored the current literature on BTIs of SARS-CoV-2 among vaccinated individuals, with a particular focus on the type of vaccine, the SARS-CoV-2 variant involved, common etiology, and immune parameters. This study aimed to identify any associated risk factors and to determine the extent to which BTIs are due to an immune evasion by VOCs as opposed to the failure of vaccines to elicit a satisfactory immune response.

## 2. Materials and Methods

### 2.1. Search Strategy and Selection Criteria

This systematic review was performed in accordance with the standards of the Preferred Reporting Items for Systematic Review and Meta-Analysis (PRISMA) Statement [10]. Approval from the Institutional Review Board was not needed. PubMed, Web of Science, ProQuest, and Embase databases were systematically searched from 2019 until 30 January 2022. A medical subject headings (MeSH) term and keyword search of each database was performed using the Boolean operators OR and AND. Keywords used included: SARS-CoV-2, SARS-CoV-2 variants, and breakthrough infections. The full search strategy for each database is provided in the Supplementary Materials.

Studies were included if they:

- Were conducted on adult patients with confirmed COVID-19 diagnosis.
- Reported COVID-19 breakthrough infections.
- Were written in the English language.
- Were peer-reviewed.
- Were either clinical trials, observational studies consisting of prospective cohort, retrospective cohort, case-control studies, case reports, or case series.
- Studies were excluded if they:
- Contained incomplete data.
- Were animal studies.
- Presented outcomes of no interest.

### 2.2. Data Extraction and Analysis

Two authors (A.I. and S.G.) independently performed the title and abstract screening. Relevant articles were then retrieved for full-text screening which was performed by two independent authors (P.S.S. and S.G.). All conflicts were resolved by a third author (A.I.). The references of the included articles were also reviewed to identify any articles missed by electronic database search.

The primary outcome of this systematic review was BTIs in vaccinated individuals; the variants causing these BTIs were also noted. The secondary outcomes were clinical and symptom severity in the vaccinated BTIs.

## 3. Results

**Figure 1:**
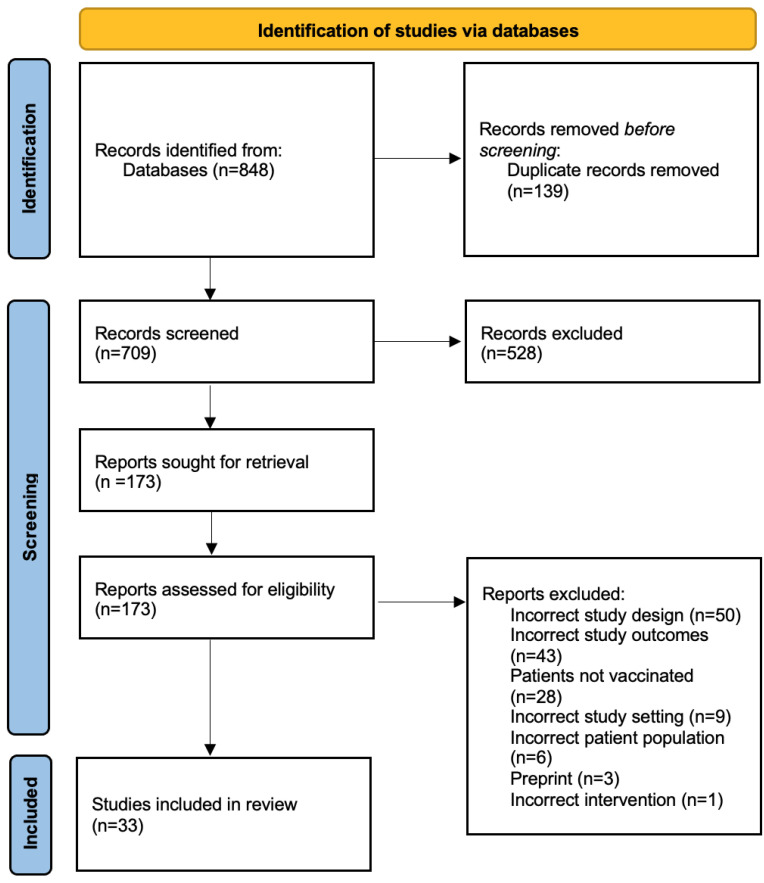
Study selection and screening following the PRISMA 2020 guidelines.

Our initial search generated 848 studies; 139 duplicates were removed; 528 studies were excluded by title and abstract screening; and 181 studies were screened for full text. We then identified 33 eligible studies describing infection with COVID-19 in those with prior vaccination (Table 1). Figure 1 depicts in detail the flow of the article selection following the PRISMA guidelines.

The total number of participants in the review who were vaccinated with two doses of vaccine was 651,595. Among these, 25,743 (3.95%) presented with BTIs. The age of the patients ranged from <15 to >83 years with a mean age of 52 years. Out of the 25,743 patients with BTIs, 11,648 (44.24%) were male and 14,068 (54.65%) were female patients. The gender of three patients was reported as “others” and the gender of 18 patients (0.07%) was unknown. BTIs presented from <4 to 185 days with a mean of 52.33 days after full vaccination (defined as completing a primary series of vaccination as recommended for the vaccine type excluding the booster).

### Study Type and Geographical Distribution

All 33 studies were observational; 19 were cohort studies, 7 were case reports, 6 were case-control studies, 1 was a longitudinal study, and 1 was a case series. The majority of the studies were conducted in the United States of America (USA) (9), followed by India (7), Italy (4), Germany (3), Israel (2), Brazil (2), Saudi Arabia, Vietnam, Mexico, Netherlands, South Korea, French Guiana, and Singapore.

Most individuals received the mRNA COVID-19 vaccinations Pfizer/BioNTech (23 studies) and Moderna (9 studies). Other vaccinations included were Covishield/AstraZeneca (10 studies), Johnson & Johnson/Janssen vaccine (4 studies), Covaxin (4 studies), Sinovac (3 studies), CanSino, and Sinopharm. Two studies did not specify which mRNA vaccine the patients received.

Among the reviewed studies, 96% of BTIs occurred with the Delta variant (B.1.617.2) and 0.94% of BTIs were due to the Alpha variant (B.1.1.7). Other variants included Gamma -P.1 (0.21%), Beta-B.1.351 (0.15%), and Kappa-B.1.617.1 (0.14%). In addition, 70 patients (0.27%) had BTIs due to non-VOCs; 19 patients were reported as other; and 17 had BTIs due to the Iota (B.1.526) variant. The serum samples of nine patients with BTIs revealed the Epsilon (B.1.427 and B.1.429) variant and four patients with the Mu (B.1.621) variant. The B.1.1.306, B.1.617.3, and 20G variants were seen in two patients each, whereas the Eta (B.1.525) and B.1.560 variants were seen in one patient each. The variant distribution for four patients was reported as unknown. Among the reported mutations, the most commonly identified were the N501Y, E484K, and the L452R mutations. Of interest, the AY.1 lineage of the Delta variant was also identified in a subset of BTIs.

A total of 8.4% of patients had pre-existing comorbidities, which included chronic bronchitis, smoking, obesity, dyslipidemia, type 2 diabetes mellitus, and immunosuppressive conditions. Moreover, 591 (2.3%) of the reported BTIs occurred in healthcare workers (HCW). The symptoms in the BTIs ranged from asymptomatic to severe pneumonia as well as intensive care unit (ICU) admission with mechanical ventilation. The majority of patients recovered without any complications. However, 11.6% of patients were hospitalized requiring oxygen supplementation, intubation, or ECMO, and 0.6% died.

## 4. Discussion

This systematic review aimed to assess the existing evidence on BTIs of SARS-CoV-2. The results shed light on the distribution of variant type, clinical outcomes, and symptom severity in BTIs, and the associative factors. SARS-CoV-2 structure and function. An understanding of BTIs begins with consideration of the characteristics of SARS-CoV-2, which comprises two groups of proteins: structural proteins (SP) and non-structural proteins (NSP). SPs are encoded by four genes, including E (envelope), M (membrane), S (spike), and N (nucleocapsid) genes [44]. NSPs are mostly enzymes or functional proteins that play a role in viral replication and methylation and may induce host responses to infection [44]. These genes are encoded in several groups, namely ORF1a (NSP1–11), ORF1b (NSP12–16), ORF3a, ORF6, ORF7a, ORF7b, ORF8, and ORF10 [44]. Importantly, not all genetic mutations lead to an increase in viral infectivity. VOCs mostly carry mutations in the spike gene, and the ORF1a frame is the critical region for mutations in the E, M, and S genes [44]. As of February 2022, over 8,600,000 sequences and eight variants of interest or concern have been identified in the global SARS-CoV-2 sequence database operated by the Global Initiative on Sharing Avian Influenza Data (GISAID) [45].

SARS-CoV-2 viral entry into the cells is facilitated by the spike protein, which attaches to the angiotensin-converting enzyme 2 (ACE2) receptor on the cell’s surface. The spike protein is split into two subunits, S1 and S2. Mutations in the S1 region, which is the receptor-binding domain (RBD) site, lower the affinity to neutralizing antibodies and show increased affinity to ACE2 receptors [46,47]. These include the N501Y (N asparagine replaced with Y tyrosine), K417N (lysine K replaced with asparagine N), and E484K (glutamic acid E replaced with lysine K) mutations in the Alpha variant. In the Beta variant, in addition to the N501Y mutation, the E484K mutations were seen, whereas both the E484K and K417T mutations were seen in the Gamma variant. The Delta and Kappa variants share the E484Q (glutamic acid E replaced with glutamine Q) and L452R (leucine L altered by arginine R) mutations. Another mutation unique to the Delta variant is T478K (threonine T replaced by lysine K) [48,49,50]. In addition to the above, mutations at the non-receptor binding site, D614G, increase the density of the spike proteins, thus leading to more functional spikes and increased replication and infectivity [51,52,53].

### 4.1. COVID-19 Vaccines and Efficacy

As of February 2022, the vaccines recommended by the World Health Organization (WHO) as part of its emergency use listing include the Comirnaty vaccine by Pfizer/BioNTech, the ChAdOx1-S nCov-19 vaccines by AstraZeneca, the Janssen/Ad26.COV 2.S vaccine by Johnson & Johnson, mRNA 1273 by Moderna, Sinopharm COVID-19 vaccine, the CoronaVac vaccine by Sinovac, BBV152 Covaxin by Bharat Biotech, Covishield (ChAdOx1-S [recombinant]) and the Covovax (NVX–CoV2373) vaccine by the Serum Institute of India, the Nuvaxovid (NVX–CoV2373) vaccine by Novavax, and the Inactivated COVID-19 Vaccine (Vero Cell) by the Beijing Institute of Biological Products [54]. The United States Food and Drug Administration (FDA) has approved three different vaccinations against SARS-CoV-2: BNT162b2 (Pfizer-BioNTech), mRNA-1273 (Moderna), and Ad26.COV2. S (Janssen) [55]. The final list of studies included these vaccines, in addition to Ad5-nCoV by CanSino, which was not yet approved for emergency use by WHO or FDA [54].

The Pfizer–BioNTech vaccine is estimated to be 90% effective after the second dose in individuals aged 80 years or older and at least 97% effective in preventing symptomatic COVID-19 cases, hospitalizations, and deaths [56]. The mRNA-1273 vaccine by Moderna is highly effective against SARS-CoV-2 after six months and has an efficacy of 94.1% against COVID-19 14 days after the first dose [57]. The Pfizer-BioNTech and Moderna vaccines contain synthetic nucleoside-modified mRNA encapsulated in lipid nanoparticles (LNP). The mRNA is translated in the cytoplasm of the cells by ribosomes into viral spike proteins activating the host immune response [58]. The AstraZeneca vaccine has a 76% efficacy in preventing symptomatic SARS-CoV-2 infection, specifically during the 15 days after the second dose (with a 29-day interval between the two doses). The vaccine utilizes an inactivated adenovirus DNA as a vector that carries the SARS-CoV-2 spike protein gene, which is then transcribed into mRNA, ultimately activating the immune system and antibody production in a manner similar to the Pfizer-BioNTech and Moderna vaccines [59]. The Sinopharm vaccine is an inactivated vaccine that stimulates the host’s immune system. It has an efficacy of 79% against symptomatic SARS-CoV-2 infection 14 days or more after the second dose (with a 21-day interval between the two doses). The Ad5-nCoV by CanSino is an adenovirus-based viral vector vaccine with an efficacy rate of 57.5% against symptomatic COVID-19 infection [60]. Ad.26.COV2.S or JNJ-78436725 Janssen vaccine is known to elicit a durable immune response for a minimum of eight months post-vaccination with minimal reductions in antibody levels [61]. The vaccine efficacy is 85.4% against critical illness and 93.1 % against hospitalization [62]. This recombinant vaccine contains an adenovirus serotype 26 (Ad26) vector that expresses a SARS-CoV-2 spike protein, which is then translated into mRNA that stimulates cellular immune responses and antibody formation against the S antigen [63]. The Sinovac vaccine is an inactivated virus vaccine, which is 51% efficacious against symptomatic SARS-CoV-2 infection, and Covaxin is an inactivated vaccine that induces a robust immune response using an adjuvant called Alhydroxiquim-II [64]. It has an efficacy of 78% against severe COVID-19 disease [64].

### 4.2. SARS-CoV-2 Variants and Breakthrough Infections

However, despite the above vaccine efficacy rates, BTIs occur. Most BTIs in our review were due to the Delta variant. This confirms the results of other studies in the literature where lowered effectiveness of the vaccines has been due to the highly transmissible Delta variant (which is 60% more transmissible than the Alpha variant) [7,64,65]. B.1.617.1 also partially impairs neutralizing antibodies elicited by BNT162b2 and ChAdOx1 nCoV-19 (Covishield) vaccines [20]. The T478K mutation in the Delta variant may also facilitate an escape by antibodies generated by vaccines or natural infection [45,66]. The AY.4 lineage of the Delta variant was seen predominantly in hospitalized patients vaccinated by the CanSino vaccine where around 67% of vaccinated individuals developed milder symptoms of COVID-19 [23]. Despite the asymptomatic or mild disease, the BTIs were associated with low levels of neutralizing antibodies, high viral load, and prolonged positivity on PCR tests, thus potentially contributing to ongoing transmission from fully vaccinated individuals [66]. Another study that analyzed the viral loads of over 16,000 infections during the predominantly Delta wave in Israel, found lower viral loads in BTIs in fully vaccinated individuals compared to infections in the unvaccinated. However, this effect started to decline after 2 months [23].

Moderate reductions in vaccine efficacy with the E484K, L452R, S477N, and N501Y mutations during the Delta variant surge were also observed in New York City between November 2020 and August 2021 [34]. However, the immune escape mutations in the spike protein gene were evenly distributed among the partially and fully vaccinated cases [34]. BTIs in which Delta was the predominant variant also revealed lowered humoral and cell-mediated immunity with Eotaxin, SCF, SDF-1a, and PIGF-1; low memory B cell cytokines (IL-1b, TNF, IFNc) and chemokines (Eotaxin, SCF, SDF-1a, PIGF-1); increased levels of plasmablast cells; and a higher frequency of CD4+ and IL-2 cells after vaccination with the BNT162b2 vaccine [42]. Compared to plasma antibodies, memory B cells were found to have a higher neutralizing effect against VOCs potentially implying that the lowered memory B cells with the Delta variant may have led to BTIs [67]. Data also shows that there is a 3-fold and 16-fold reduction in neutralization against the Delta and Beta variants as compared with the Alpha variant with BNT162b2 vaccinated sera, and a 5-fold and 9-fold reduction against the same with ChAdOx1 nCoV-19 [68].

The N501Y mutation predominantly seen in the studies yielded by our review also lowers the neutralization capacity of the vaccines [25,69]. Infections with the N501Y mutation in the Alpha variant led to low neutralizing antibodies against the AZD1222 vaccine compared to non-Alpha variants [14].

Similarly, both the E484K and S477 mutations, found in P.1 and P.6 respectively, are reported to escape neutralization by a range of mAbs [70]. E484K is also associated with a decrease in the neutralizing activity of convalescent and post-vaccination (BNT162b2) sera [71,72,73]. E484K causes resistance to many class 2 RBD-directed antibodies, including bamlanivimab [74,75]. The most potent mRNA vaccine-elicited monoclonal antibodies were over 10-fold less effective against pseudotyped viruses carrying the E484K mutation [18]. In the study by Olsen et al., BTIs in fully vaccinated patients due to the E484K variant mutations in the Alpha variant had a significantly lower cycle threshold (a proxy for higher virus load) and significantly higher hospitalization rate [40]. Other variants (e.g., B.1.429 and B.1.427, P.1, P.2 (Zeta), and R.1) also increased rapidly, although the magnitude was less than that in Alpha [40]. Additionally, patients infected with the B.1.617.1 or B.1.617.2 variants also had a high rate of hospitalization despite vaccination^51^. In addition to the above, the L452R mutation, where Leucine-452 that is located at the point of interaction with the ACE2 receptor in the RBD receptor is replaced by arginine, also causes greater receptor affinity and escape from neutralizing antibodies [20,24,76].

Although most BTIs reported in the final 33 studies occurred before full vaccine-induced immunity, a few reinfections were also reported despite the presence of neutralizing antibodies [28]. Schulte et al. reported the case of an HCW who developed infection with the Eta (B.1.525) variant despite the presence of neutralizing antibodies seven weeks after vaccination [77]. The authors hypothesized that this could be attributed to the absence of an N-specific antibody and spike-based neutralization post-vaccination, which prevents antibody responses to the nucleocapsid, thus demonstrating the need for protective measures such as masks even after full vaccination [78]. As per their study, neutralization assays demonstrated differences against variants by a factor of 4. Variant B.1.525 is the best at neutralizing, followed by the B.3 and B.1.1.7 variants. The B.1.351 variant neutralizes the least. The study concluded that differences in spike proteins play a crucial role in neutralization [78]. Another study showed similar results, with higher neutralization against B.1.525 and B.1.1.7 and weaker neutralization against B.1.351 compared to B.1 [79].

### 4.3. Breakthrough Infections in at-Risk Populations

#### 4.3.1. Immunosuppression

Laboratory and clinical investigations among the final 33 studies showed that post-vaccine antibody responses against SARS-CoV-2 variants are less than antibody responses against wild SARS-CoV-2 but are still protective against severe disease and death [80,81]. This phenomenon is applicable for immunocompetent patients who are mounting high antibody responses that can overcome the mutations in the spike protein but inadequate for solid organ transplant recipients and those with immunosuppression who mount a suboptimal antibody response against wild SARS-CoV-2 [82]. In patients with solid organ transplantation, lower antibody response and waning immunity render those patients at higher risk of BTIs after vaccination. In addition, immunosuppressive medications such as calcineurin inhibitors, mycophenolic acid, and antiproliferative drugs were reported to increase the risk of SARS-CoV-2 BTIs by lowering the immunogenicity of vaccines and in developing an adequate immune response [17,83].

In a study by Deng et al., BTIs occurred in fully vaccinated individuals over four weeks of follow-up [76]. Fourteen patients were identified and 42.8% were solid organ transplant (SOT) recipients. Another study by Almaghrabi et al. demonstrated that BTIs after COVID-19 mRNA vaccination were highest in immunocompromised patients with primary immunodeficiencies, active malignancies, and transplantation [84]. In one study, patients with cancer undergoing chemotherapy had lower levels of antibodies compared to healthy controls following the second dose of the BNT162b2 vaccine [43]. Sun et al. demonstrated that full vaccination was associated with a reduced rate of BTIs regardless of the immune status [85]. However, even among these, the rate of BTIs was still higher in the immunocompromised group thus necessitating the need for alternate strategies such as monoclonal antibodies and non-pharmaceutical personal protective measures such as masks, social distancing, and avoiding large gatherings [85]. Immunosuppressed individuals also had a higher risk factor for BTIs when controlled for age, gender, and comorbidities [85]. To combat this, the third dose of the vaccine was initially recommended for immunocompromised patients [86]. However, studies still revealed a substantially lower immune response compared to the general population, thus paving the way for treatment with monoclonal antibodies [87,88].

#### 4.3.2. Aging

Our study revealed that the aging of the immune system or immunosenescence, which decreases the number of naive T & B cells, can also lead to reduced vaccine efficacy, particularly in older individuals, thus predisposing them to BTIs [84,89]. A recent study that described humoral and cell-mediated responses after two doses of mRNA vaccination against SARS-CoV-2 VOCs in relation to different age groups showed that patients above eighty years old had lower cell-mediated responses compared to younger patients [90]. Another multicenter study in the USA that examined the factors affecting COVID-19 immunity in individuals who were administered two doses of the BNT162b2 vaccine, found that antibody titers were negatively correlated with increasing age [11]. Sun et al. who analyzed the risk of BTIs in immunocompromised patients, found that although full vaccination was associated with a 28% reduced risk of BTIs, older individuals still had a higher rate of BTIs [85].

#### 4.3.3. Occupational Risk

Lastly, the results showed that reinfections were seen due to prolonged exposure, predominantly in healthcare workers despite vaccination [16,20,29,31,41,69,91]. Although occupational exposure other than healthcare settings was not reported in the studies in our review, prolonged exposure to COVID-19 has also been known to occur in retail workers, meat and poultry workers, shelter staff, call center staff, and transit operators [92]. As per the WHO prior to the availability of COVID-19 vaccines, HCWs accounted for 14% of COVID-19 cases [93]. Several studies have also reported milder infection in HCWs, and this could be due to the availability of frequent testing and detection [94]. Although our review reported no comorbidities among HCWs, around 6% of HCWs in previous studies who presented with severe infection had comorbidities such as obesity [94]. The risk of BTIs among HCWs is said to have declined after the introduction of COVID-19 vaccinations, with a greater proportion of infections from community exposure. Despite this, BTIs due to waning immunity and the emergence of variants still present a risk to patients and coworkers, highlighting the need for ongoing screening and testing in this population [95].

#### 4.3.4. Ct (Cycle Threshold) Values & Viral Loads

The Ct (cycle threshold) value is the number of cycles it takes for the RT-PCR test to detect the virus. Ct levels are inversely proportional to the amount of target nucleic acid in the sample. The higher the amount of the viral nucleic acid in the sample, the lower the Ct value. An important issue for controlling the spread of variants is to determine if the BTI is associated with high viral loads that may result in a secondary spread. Previous studies reported that low viral loads and a high Ct value were detected following vaccination [23,96]. In contrast, a study by Deng et al. detected relatively high viral loads (median Ct of 19.6) even in non-immunosuppressed vaccinated subjects exhibiting asymptomatic or mild infection [28]. This finding is consistent with other studies that reported that individuals with BTIs with the Alpha variant had a significantly lower Ct value compared to non-Alpha patients [40]. Although this could be viewed as an enhanced transmissibility potential of Alpha, no clear correlation between Ct values and transmission rates has been confirmed.

#### 4.3.5. Heterogenous Vaccination Regimens

Numerous studies have shown a stronger immune response where mix and match vaccine regimens are used [19,97,98]. Individuals who receive different types of COVID-19 vaccines for their first, second, and subsequent booster doses show more potent immune responses. One study in our review described the transmission of infection from a fully vaccinated spouse, thus hypothesizing that this was due to a lack of immune response against the nucleocapsid protein, against which the mRNA vaccines are not effective. A study by Nordstrom et al., found that those who received a mixed vaccine regimen were 68% less likely to develop an infection compared to unvaccinated people, whereas those who received two doses of the same vaccine (Astra Zeneca) were 50% less likely to do so [82]. Another study also showed similar results where the vaccine efficacy against SARS-CoV-2 infection was 88% when ChAdOx1 and an mRNA vaccine were combined [83]. Additionally, there is some evidence that heterologous vaccination may also confer greater protection, with combined cellular and humoral immunity in immunocompromised individuals [84].

## 5. Limitations

Importantly, our study has several notable limitations. Given the nature of surveillance, testing, and reporting, oftentimes not all cases are documented. There may also have been some overlap in status (e.g., some individuals who had been vaccinated may have been previously infected at some point). We describe the cases that have been documented in the scientific literature. Additionally, we must consider the possibility of asymptomatic viral transmission among vaccinated individuals; these numbers are not reflected in these studies. Thus, it could be possible that the extent of SARS-CoV-2 transmissibility among vaccinated individuals is greater than expected as per our current understanding. Data reported from hospital settings where exposure to infection is higher, may not reflect the infection rates in the general population. Also, data in several studies were collected from electronic medical records and hence may be prone to error. Similarly, the history of exposure in those with BTIs may not always be accurate and the source of infection is not always known. Among the data from the immunocompromised patients, there were no specific mentions of which condition may have had a greater contribution towards the lowered immunity.

## 6. Conclusions

BTIs remain a critical challenge in controlling the epidemic. Whether individuals with BTIs contribute substantially to the onward transmission of SARS-CoV-2 in the population currently remains unclear. In our review, we found that BTIs do not reflect selection towards specific immunity-evading variants, rather, they reflect the most prevalent variant in the community at that time. Hence a standardized surveillance reporting protocol for suspected BTIs is necessary to better assess the nature and extent of the burden of reinfections in vaccinated individuals. Studies on BTIs could be helpful to understand the neutralizing response to SARS-CoV-2 infection and the corresponding immunity. However, the absence of systematic genomic sequencing of positive cases worldwide impedes advances in public health surveillance to manage the pandemic at the individual and collective levels. Further investigations, including a genetic comparison of SARS-CoV-2 strains, would be beneficial to understanding the frequency and pathophysiology of SARS-CoV-2 reinfections. Although COVID-19 vaccines have proven to be highly effective, the possibility of BTIs remains a reality, particularly in the context of emerging variants of concern. Many factors contribute to BTIs including the transmission dynamics of SARS-CoV-2 variants and their biological capacity to survive, behavioral characteristics of individuals, and vaccination status. Future studies should explore the role of combining different types of vaccines, post-exposure prophylaxis, and close monitoring for disease progression including disease progression and transmission in high-risk individuals such as HCWs or immunocompromised patients.

## Figures and Tables

**Table 1:** Included studies and patient characteristics of reinfection in vaccinated individuals.

Study	Type of Study	Number of Fully Vaccinated Individuals (Breakthrough Infections)	Country	Gender	Age (Years)	Number of Days since Vaccination	Vaccine Received	Symptoms	Comorbidities	Variants [Reported Mutations]	Ct (Cycle Threshold) Value	Complications & Outcome
Bergwerk et al., 2021 [11]	Case control	11,453 (39)	Israel	Females: 25; Males:14	Mean: 42	Median: 39 (Range: 11–102)	BNT162b2 (Pfizer-BioNTech)	Upper respiratory congestion (36%), myalgia (28%), loss of smell or taste(28%); fever or rigors (21%); Asymptomatic (33%)	Immunosuppressed (1), CLL*(1), ITP*(1), metabolic syndrome (6), thyroid disorder (3), other (migraines, fibromyalgia, osteoporosis, PCOS*) (4)	Alpha (B.1.1.7): 85% of samples	<30 (74%); >30 (26%)	Recovery
Estofolete et al., 2021 [12]	Case Report	2 (2)	Brazil	Male	60	106	Corona Vac (Sinovac)	Anosmia, malaise, myalgia, dyspnea	Type 2 diabetes mellitus, hypertension, obesity degree I (BMI*: 32.3 kg/m^2^)	Gamma (P.1) [K417T, E4844K, N501Y]	Unknown	Hospitalization with supplemental oxygen → Recovery
					55	122		Sore throat, headache, malaise, chills, coryza, sneezing, dyspnea, hypoxia	None			
Fabiani et al., 2021 [13]	Case Report	1 (1)	Italy	Male	83	23	BNT162b2(Pfizer-BioNTech)	Slight headache, mild cold	None	Gamma (P.1) [K417T, E484K, N501Y, D614G]	13	Recovery
Philomina et al., 2021 [14]	Retrospective cohort	6 (6)	India	Female	25	35	AZD1222/Covishield (SII)	Influenza-like illness	Unknown	B.1.1.306 [E484K]	16.45	Recovery
				Male	50	30		Fever, malaise, anosmia, headache		Alpha (B.1.1.7) [N501Y]	20	
				Female	53	28		Rhinitis			21	
					25	26		Fever, loose stools, abdominal pain, dry cough, myalgia, rhinitis, anosmia			24	
					32	25		Mild nasal congestion, headache			26	
					33	17		Loss of smell, loose stools, rhinitis		B.1.1 B.1.560 [S477N]	14	
Hacisuleyman et al., 2021 [15]	Prospective cohort	417 (2)	USA	Female	51	19	mRNA-1273 (Moderna)	Sore throat, congestion, headache, anosmia	None	Alpha (B.1.1.7) [E484K D614G T95I, del142–144]	24.2	Recovery
					65	36	BNT162b2 (Pfizer–BioNTech)	Fatigue, sinus congestion, headache		Alpha (B.1.1.7) [S477N T95I, del142–144 R190T F2201 R237K R246T D614G]	33.3	
Kroidl et al., 2021 [16]	Case report	1 (1)	Germany	Unknown	Early ‘60s	26	BNT162b2 (Pfizer–BioNTech)	Headache, congested nose	None	Beta (B.1.351)	Unknown	Recovery
Almaghrabi et al., 2022 [17]	Case series	4 (4)	Saudi Arabia	Male	68	73	BNT162b2 (Pfizer-BioNTech)	Fever, chills, vomiting	Liver transplant, diabetes mellitus, hypertension, immunosuppressive medication	Alpha (B.1.1.7) [E484K]	25	Severe pneumonia →Mechanical ventilation →Death
					69	150		Shortness of breath, hypoxia	Renal transplant, diabetes mellitus, hypertension, immunosuppressive medication	Alpha (B.1.1.7)	30	Pneumonia → Mechanical ventilation, septic shock →Death
					41	39		Mild coughs, shortness of breath	Renal transplant, immunosuppressive medications	Beta (B.1.351)	29	ICU* admission with HFNC*→ Recovery
				Female	48	21	ChAdOx1 nCoV-19 vaccine (AstraZeneca)	Fever, hypoxia	Renal transplant, post-transplant lymphoma, immunosuppressive medications	Delta (B.1.617.2)	19	Hospital acquired infection, HFNC*→ Recovery
Baj et al., 2021 [18]	Retrospective cohort Study	4 (4)	Italy	Female	80	77	mRNA-1273 (Moderna)	Fatigue, headache, myalgia, dyspnea	Unknown	Delta (B.1.617.2)[E484K]	22	Recovery
				Male	77	67	Pfizer-BioNTech (BNT162b2)	Fever			19	
				Female	83	87	BNT162b2 (Pfizer-BioNTech)	Fever, fatigue, ageusia, anosmia			18	
				Female	81	45	mRNA-1273 (Moderna)	Dyspnea, fever, myalgia, fatigue			21	Hospital admission → Recovery
Bignardi et al., 2022 [19]	Case report	1 (1)	Italy	Male	61	120	mRNA vaccine -type not specified	Dyspnea, cough, fever	Hypertension, obesity	Delta (B.1.617.2)	Unknown	Pneumonia → Death
Chau et al., 2021 [20]	Cohort	866 (62) ^a^	Vietnam	Females: 29 Males:33	Median: 41.5 (IQR: 32–50)	49–56 (97%)	Oxford-AstraZeneca	Fever (27%), cough (37%), sore throat (34%), runny nose (36%), loss of smell (39%), loss of taste (8%), muscle pain (27%), headache (19%), chest pain (3%), nausea (8%), shortness of breath (4%), pneumonia (5%), asymptomatic (21%)	Overweight (6), obesity (3), hypertension (3), hepatitis B (3), diabetes mellitus (2), pregnancy (1)	Delta (B.1.617.2)	31.9 (IQR: 23.3–34.9)	Recovery
Connor et al., 2021 [21]	Case report	2 (2)	USA	Male	63	60	BNT162b2 (Pfizer-BioNTech)	Nasal congestion, headache, dry cough	Hypertension, benign prostatic hypertrophy, overweight	B.1.617.2 B.1.619 [35 mutations detected, including 9 in the spike protein]	31.3	Recovery
					25	90		Upper respiratory symptoms, headaches	None	B.1.617.2 [7/12 shared S-gene mutations]	25.2	Recovery
Gharpure et al., 2022 [22]	Cohort	1128 (918)	USA	Females: 90 Males: 822	19–49 (66%), 50–64 (30%), 65–74 (4%), >75 (0.4%)	>14	BNT162b2 (Pfizer-BioNTech): 504 mRNA-1273 (Moderna): 293 Johnson & Johnson: 121	Abdominal pain (6%), chills (35%),congestion (58%),cough (73%), diarrhea(20%), shortness of breath (10%), fatigue (41%), fever (43%), headache (47%), loss of appetite (16%), loss of smell or taste (50%), muscle pain (39%), sore throat (42%), vomiting (3%)	Active cancer (3), autoimmune disease (11), cardiovascular disease (36), chronic kidney disease (3), chronic lung disease (22), pregnancy (3), diabetes mellitus (21), HIV* infection (6), solid organ transplant (1), other immunosuppressive conditions (41)	Delta (B.1.617.2): 98%Delta (AY.3 sublineage:0.3%, Delta (AY.4 sublineage): 0.8% Gamma (P.1): 0.8%	Unknown	Hospitalized (7), ICU* (2) → Recovery
Galan Huerta et al., 2022 [23]	Case-control	53 (53)	Mexico	Females: 28; Males: 25	Mean: 59.7 (50–70)	7	AstraZeneca/Oxford: 8 (15%) BNT162b2 (Pfizer/BioNTech): 8 (15%) Convidecia(CanSino): 24 (45%) CoronoVac (Sinovac): 10 (19%) Unspecified: 3	Mostly mild or asymptomatic	Hospitalized: hypertension (11), Type 2 diabetes mellitus (13), obesity (1), smoking (4); Ambulatory: hypertension (5), Type 2 diabetes mellitus (5), obesity (2), smoking (1)	Delta (B.1.617.2) (AY.1, AY.2, AY.3, AY.4 lineage): 67.92% Gamma (P.1, P.1.1, P.1.2): 7.55% Mu (B.1.621): 7.55% Alpha (B.1.1.7): 5.66%	Hospitalized: 19.58 (17.19–22.49); Ambulatory: 18.81 (15.72–21.24)	Hospitalized: High-flow O2 (14), intubation (10), ICU* admission (1), death (4); Ambulatory: all recovered (30)
Deng et al., 2021 [24]	Case control	14 (14)	USA	Female	60	Range: (14–109)	BNT162b2 (Pfizer-BioNTech)	Rhinorrhea	None	Alpha 20I/S: 501Y.V1	18.8	Recovery
				Male	58			Chill, subjective fever	None	Alpha (20I/S: 501Y.V1)	19.1	
				Female	48			Weakness, congestion loss of taste/smell, fatigue	Smoker	Alpha (20I/S: 501Y.V1)	20.9	
					51		mRNA vaccine type not provided	Headache, cough, rhinorrhea ageusia, anosmia	Immunosuppressive medication, non-alcoholic steatohepatitis	Gamma (20J/S: 501Y.V3)	17.1	
					37		mRNA-1273 (Moderna)	Asymptomatic	None	20G	19.5	
					50		BNT162b2 (Pfizer-BioNTech)	Asymptomatic	None	Unknown	34.2	
					81		Johnson & Johnson	Shortness of breath, cough	Heart disease, cerebrovascular disease	Alpha (20I/S: 501Y.V1)	18.8	Hospitalization → recovery
				Male	65		BNT162b2 (Pfizer-BioNTech)	Diarrhea, myalgia, chills, fever	Immunosuppressive medication, kidney, and heart transplant	Alpha (20I/S: 501Y.V1)	20.1	Pneumonia → recovery
					55			Cough, acute hypoxic respiratory failure, sepsis	Immunosuppressive medication, kidney transplant	Alpha (20I/S: 501Y.V1)	22.3	Intensive Care Unit (ICU) → Death
					70			Cough, weakness, fever, dyspnea	Immunosuppressive medication, liver transplant	Gamma (20J/S: 501Y.V3)	19.6	Hospitalization → recovery
					68		mRNA-1273 (Moderna)	Acute hypoxia, acute pneumonia, hemoptysis	Immunosuppressive medication, lung transplant	Gamma (20J/S: 501Y.V3)	21.4	
				Female	60			Shortness of breath, fever, chills, body aches, hypoxia	Immunosuppressive medication, lung transplant	Gamma (20J/S: 501Y.V3)	15.7	Intensive Care Unit (ICU) → Recovery
				Male	65		BNT162b2 (Pfizer-BioNTech)	Diarrhea, nausea, weakness cough, dyspnea	Immunosuppressive medication, liver transplant	Epsilon (CAL.20C)	22.1	Hospitalization → Recovery
				Female	76			Fever, chills, acute respiratory failure	None	20G	18.3	Intensive Care Unit (ICU) → Recovery
De Souza et al., 2021 [25]	Case control	42 (22)	Brazil	Females: 17 Males: 5	77 (IQR: 51–87)	5–27	CoronaVac (SinoVac)	Asymptomatic (75%) Mild COVID-19 symptoms (25%)	Unknown	Alpha (B.1.1.7)	Unknown	Death: 1% Recovery: 99%
Gupta et al., 2021 [26]	Case control	592 (592)	India	Females: 207; Males: 385	Mean 44 (31–56)	39 (19–58)	Covaxin: 71 (10.5%)Covishield (AstraZeneca): 604 (89.2%) Covilo (Sinopharm): 2 (0.3%)	Symptomatic (71%) with one or more symptoms, fever (69%), body ache, headache and nausea (56%), cough (45%), sore throat (37%), loss of smell and taste (22%), diarrhea (6%), breathlessness (6%), ocular irritation, redness (1%); Asymptomatic (29%)	Type 2 diabetes mellitus, hypertension, obesity, chronic cardiac, renal, and pulmonary diseases	Delta (B.1.617.2): 384 Alpha (B.1.1.7): 28) Kappa (B.1.617.1: 22 B.1.617.3: 2 B.1.36: 2 B.1.1.294: 1 B.1.36.16: 1 B.1.1.306: 1 Delta (AY.2): 2	<30	Fully vaccinated: hospitalized (53), Recovered (589), Death (3)
Kale et al., 2021 [27]	Cohort	1639 (156)	India	Female: 86 Males: 70	Median: 34 (IQR21–67)	>14	ChAdOx1 nCoV-19/Covishield (SII)	Fever, muscle aches	Unknown	Delta (B.1.617.2): 32 Kappa (B.1.617.1):11 Alpha (B.1.1.7): 1	23.2 (IQR 0.0–33.1)	Recovery; Hospitalization (0.22%)
Schulte et al., 2021 [28]	Case report	1 (1)	Germany	Male	42	49	BNT162b2 (Pfizer-BioNTech)	Asymptomatic	None	B.1.525	9.44	Recovery
Malhotra et al., 2022 [29]	Retrospective cohort	1079 (17)	India	Unknown	<25:72 (6.6%); 25–44: 660 (60.6%);≥ 45: 357 (32.8%)	>15	BBV152/Covaxin (Bharat Biotech)	Symptomatic:-Fever, rhinorrhea, sore throat, cough, chest pain, wheezing, difficulty breathing, shortness of breath, anosmia, dysgeusia, fatigue, myalgia, headache, abdominal pain, nausea, diarrhea. Asymptomatic: 3	Hypertension; chronic heart, lung, or kidney disease; cancer; hypothyroidism	Gamma (B.1.617.2)	Unknown	Recovery
Shastri et al., 2021 [30]	Case report	1(1) ^b^	India	Female	61	28	ChAdOx1 nCoV-19(Covishield)	1st infection episode: Abdominal pain, fever, myalgia, fatigue 2nd infection episode: Body ache, fatigue, headache, cough, breathlessness, fever, rhinorrhea, vomiting	Prediabetes, bronchial asthma, hypertension	Alpha (B.1.1.7):1st Delta (B.1.617.2):2nd	1st infection: 35.2 2nd infection: 20.4	Recovery
Rovida et al., 2021 [31]	Cohort	3702 (33)	Italy	Females:26 Males: 7	Unknown	47 (Range 7–90)	BNT162b2 (Pfizer-BioNTech)	Asymptomatic (48%), fever (6%), asthenia (6%), headache (6%), arthralgia (9%), pharyngodynia (3%), rhinitis (27%), cough (9%), cough (9%), anosmia (9%), ageusia (3%), nausea (9%), diarrhea (10%)	Unknown	Alpha (B.1.1.7)	Unknown	Recovery
Rumke et al., 2022 [32]	Cohort	14 (14)	Netherlands	Female	45	43	BNT162b2 (Pfizer-BioNTech)	Anosmia, arthralgia, fever, headache, myalgia, peripheral neuropathy, rhinosinusitis	None	Alpha (B.1.1.7)	18.5	Recovery
					62	78		Anosmia, rhinosinusitis	None	Alpha (B.1.1.7)	23.7	
					27	64		Rhinitis	None	Alpha (B.1.1.7) [A771V]	23.5	
					52	61		Cough, dyspnea, fever	Asthma	Alpha (B.1.1.7)	19.6	
					35	74		Anosmia, cough, rhinitis	None	Alpha (B.1.1.7) [H245Y]	21.5	
					35	80		Anosmia, rhinosinusitis	None	Alpha (B.1.1.7) [S494P]	24.7	
				Male	58	80		Asymptomatic	None	Alpha (B.1.1.7)	31.9	
				Female	26	111		Fever, rhinitis	None	Alpha (B.1.1.7) [V382L]	19.8	
					38	38	Ad26.COV2.S (Johnson & Johnson)	Cough, fever, pharyngitis, rhinosinusitis	None	Alpha (B.1.1.7) [D88V]	18.9	
					57	37		Cough, dyspnea	None	Alpha (B.1.1.7) [V483I, A706V]	21.5	
					50	20		Asymptomatic	None	Alpha (B.1.1.7) [S12F, D905N]	23.6	
					54	45		Cough, fever, headache, myalgia, otitis	Atopic dermatitis	Delta (B.617.2)	31.3	
					38	18		Asymptomatic	Hashimoto thyroiditis	Alpha (B.1.1.7)	29.0	
					48	52		Anosmia, fever, headache, myalgia, sinusitis	None	Delta (B.617.2) [G142D]	29.2	
Yi et al., 2022 [33]	Cohort	24 (24)	South Korea	Females:18	78.9 (Range 34–99)	Mean: 40 (Range, 80–117)	BNT162b2 (Pfizer-BioNTech)	Asymptomatic (48%) Symptomatic (48%)	Unknown	Delta (B.1.617.2):13	18.1 (symptomatic); 20 (asymptomatic)	Recovery (96%), Death (4%)
Robilotti et al., 202 [34]	Cohort	12,046 (80: pre-Delta)	USA	Females: 60 Males: 20	Median37 (Range:22–65)	Median: 56 (Range:1–100)	BNT162b2 (Pfizer-BioNTech): 91% mRNA-1273 (Moderna): 9%	Asymptomatic (20%) Headache (55%) Fatigue (45%) Body aches (28%) Fever (including subjective) (19%) Loss of smell/taste (28%) Chills (20%) Sore throat (21%) Rhinorrhea, nasal congestion, sneezing (53%) GI symptoms (nausea, vomiting, diarrhea or abdominal pain) (18%) Cough (31%) Shortness of breath (8%)	Unknown	Alpha (B.1.1.7) [E484K K417T/N S477N N501Y]	Unknown	Recovery
		(179: post-Delta)		Females: 127 Males: 52	Median 33 (Range 21–63)	Median: 185 (Range 8–235)	BNT162b2 (Pfizer-BioNTech): 79% mRNA-1273 (Moderna): 21%	Asymptomatic (8%) Headache (45%) Fatigue (55%) Body aches (37%) Fever (including subjective) (32%) Loss of smell/taste (28%) Chills (27%) Sore throat (44%) Rhinorrhea, nasal congestion, sneezing (52%) GI symptoms (nausea, vomiting, diarrhea or abdominal pain) (17%) Cough (53%) Shortness of breath (9%)	Unknown	Delta (B.1.617.2) [L452R T478K E484Q]	Unknown	Recovery
Vignier et al., 2021 [35]	Cohort	25 (15)	French Guiana	Males: 15	Median: 53.3	>14	BNT162b2 (Pfizer-BioNTech): 56.8%	Symptomatic: Fever, dyspnea (87%)	Hypertension, diabetes mellitus, obesity, cardiac insufficiency	Gamma (P.1)	18–35	Recovery
Tober-lau et al., 2021 [36]	Longitudinal	20 (16)	Germany	Females: 12 Males: 4	>65 years	4–5	BNT162b2 (Pfizer-BioNTech)	Asymptomatic mostly. Diarrhea, fatigue, cough or shortness of breath (31.25%)	Hypertension, Type 2 diabetes mellitus, chronic kidney disease dementia	Alpha (B.1.1.7)	Unknown	Hospitalization (31.25%) Supplemental oxygen (6.3%) Death (12.5%)
Servellita et al., 2022 [37]	Cohort	1373 (125) ^c^	USA	Females: 68 Males: 57	Mean: 49 (Range 22–97)	Median: 73.5 (range 15–140)	BNT162b2 (Pfizer-BioNTech): 51%, mRNA-1273 (Moderna): 31% Johnson & Johnson: 10%	Asymptomatic (26%) COVID-19 pneumonia (15.4%)	Immunocompromised (23%)	Delta (B.1.617.2:31%, Alpha (B.1.1.7): 18.3%, Gamma (P.1): 15.6%, Iota (B.1.526): 11.9%, Epsilon (B.1.427/B.1.429): 6.4%, Beta (B.1.351): 3.7%, Other: 12.8% [L452R/Q, E484K/Q and/or F490S]	23.1	Recovery (100%) ICU (2.6%), Hospitalizations (15.4%)
Singer et al., 2021 [38]	Prospective cohort	343 (31)	Israel	Females: 17 Males: 14	Median: 58 (21–87)	>7	BNT162b2 (Pfizer-BioNTech)	Asymptomatic (05%)	Unknown	Beta (B.1.351)	Unknown	Recovery
Thangaraj et al., 2022 [39]	Prospective cohort	113 (113)	India	Females: 44 Males:66 Others:3	Median:54 (42–64)	>14	Covaxin: 27.4% Covishield: 70.8% Unknown: 1.8%	Symptomatic (88.5%)	Unspecified comorbidities (46%)	Delta (B.1.617.2):74.3% B.1.617.1: 0.9% AY.1: 0.9% Alpha (B.1.1.7): 0.9% Beta (B.1.351): 0.9%	<30	Recovery
Olsen et al., 2021 [40]	Cohort	12,476 (207)	USA	Females: 53% Males: 47% ^d^	Median: 52.5 ^d^	>14	BNT162b2 (Pfizer-BioNTech): 87% mRNA-1273 (Moderna): 13%	Unknown	BMI > 30 (42.7%)	Alpha (B.1.1.7): 126; Gamma (P.1): 5 Epsilon (B.1.429): 3 B.1526: 1 B.1526.1:1 Eta (B.1.525): 1 non-VOC: 70	23.9	Hospitalization (34.8%)
Singh et al., 2022 [41]	Cohort	63 (36)	India	Females: 13 Male: 23	Median: 37 (21–92)	Unknown	AZD1222/Covishield (SII): 15.87% BBV152/Covaxin: 84.13%	High-grade unremitting fever, shortness of breath, headache	None	Delta (B.1.617.2): 63.9% B.1.617.1: 11.1% Alpha (B.1.1.7) 2.8%	Range: 11.3–31	Recovery
Tay et al., 2022 [42]	Prospective case-control	55 (55)	Singapore	Females: 19 Males: 36	Median 46 (IQR 36.5–59.5)	82 (IQR 51.5–99)	BNT162b2 (Pfizer-BioNTech)	Asymptomatic (21.8%) Mild symptoms (78.2%)	Chronic venous, asthma, other chronic lung diseases, rheumatologic disease, chronic liver disease, diabetes mellitus, chronic kidney disease, malignancies, or HIV (6)	Delta (B.1.617.2): 87.3% Unknown: 7.3% Non-Delta:5.5%	Unknown	Recovery
Sun et al., 2021 [43]	Retrospective cohort	604,035 (22,917)	USA	Females: 13,040 Males: 9877	Median: 51 (IQR 34–66)	138 (85–178)	BNT162b2 (Pfizer-BioNTech) mRNA-1273 (Moderna)	Unknown	Immunocompromised (1451).	Delta (B.1.617.2)	Unknown	Recovery (93.5%); Hospitalization: 11.5% Severe outcomes (0.65%)

* Abbreviations: CLL—Chronic Lung Disease; ITP—Idiopathic thrombocytopenic purpura; PCOS—Polycystic ovarian syndrome; BMI—Body mass index; HIV: Human Immunodeficiency Virus; ICU: Intensive Care Unit; HFNC: High flow nasal cannula. ^a^ Only 62 participants included in the study; ^b^ Patient had 2 breakthrough infections; ^c^ Variant breakdown provided for 109 patients; ^d^ Patient data corresponds to total number of patients.

## Supplementary Materials

The following are available online at https://www.mdpi.com/article/10.3390/tropicalmed7050081/s1, The full search strategy for each database.
