# Supplementary material for: Characteristics of COVID-19 Breakthrough Infections among Vaccinated Individuals and Associated Risk Factors: A Systematic Review
Source: Trop Med Infect Dis. 2022 May 22;7(5):81. doi: 10.3390/tropicalmed7050081 (PMC9144541; doi:10.3390/tropicalmed7050081)
Supplement: Supplementary file 1 [file tropicalmed-07-00081-s001.zip › tropicalmed-1709003-supplementary.pdf]

COVID-19 breakthrough infections among vaccinated individuals: characteristics, variant distribution, and associative risk factors

Date: 1/30/2022

PubMed

CONCEPT 1:

("SARS-CoV-2 variants" [Supplementary Concept] OR ("SARS-CoV-2"[Mesh] AND variants) OR “Sars CoV-2 variants”)

Results: 3,824

CONCEPT 2:

(“202012-01" OR ("20A" AND "S484K") OR ("20B" AND "S265C") OR "20E" OR "20h 501y v2" OR "614G" OR "A.1.177" OR "A.23.1" OR "alpha" OR "B.1.1.7" OR "B.1.351" OR "B.1.427" OR "B.1.429" OR "B.1.525" OR "B.1.526" OR "B.1.617.1" OR "B.1.617.2" OR "beta" OR "C.37" OR "CAL.20C" OR "cluster 5" OR "D614G" OR "delta" OR "E484K" OR "epsilon" OR "gamma" OR "iota" OR "kappa" OR "L452R" OR "lambda" OR "mink-associated" OR "P.1" OR "P.2" OR "P.3" OR "theta" OR "zeta")

Results: 2,854,174

CONCEPT 3:

("breakthrough infections" OR ((breakthrough OR breakthroughs) AND ("infections"[MeSH Terms] OR infections)) OR "Reinfection"[MeSH Terms] OR reinfection)

Results: 18,886

FINAL: #1 AND #2 AND #3: 140

Embase

CONCEPT 1:
'coronavirus disease 2019'/exp

187,945

CONCEPT 2:

'sars-cov-2 variant 501y.v2'/exp OR 'sars-cov-2 variant 501y.v1'/exp OR 'sars-cov-2 (germany/fi1103201/2020 (epi-isl 463008, d614g))'/exp OR '20a' OR 'sars-cov-2 variant s484k' OR 'sars-cov-2 variant 20b' OR 'sars-cov-2 variant s265c' OR 'sars-cov-2 variant 20e' OR 'sars-cov-2 variant 20h 501y v2' OR 'sars-cov-2 variant 614g' OR 'sars-cov-2 variant a.1.177' OR 'sars-cov-2 variant a.23.1' OR 'sars-cov-2 variant alpha' OR 'sars-cov-2 variant b.1.1.7'/exp OR 'sars-cov-2 variant b.1.351'/exp OR 'sars-cov-2 variant b.1.427' OR 'sars-cov-2 variant b.1.429'/exp OR 'sars-cov-2 variant b.1.525' OR 'sars-cov-2 variant b.1.526' OR 'sars-cov-2 variant b.1.617.1' OR 'sars-cov-2 variant b.1.617.2' OR 'sars-cov-2 variant beta' OR 'sars-cov-2 variant c.37' OR 'sars-cov-2 variant cal.20c'/exp OR 'sars-cov-2 variant cluster 5' OR 'sars-cov-2 variant d614g' OR 'sars-cov-2 variant delta' OR 'sars-cov-2 variant e484k' OR 'sars-cov-2 variant epsilon' OR 'sars-cov-2 variant gamma' OR 'sars-cov-2 variant iota' OR 'sars-cov-2 variant kappa' OR 'sars-cov-2 variant l452r' OR 'sars-cov-2 variant lambda' OR 'sars-cov-2 variant mink-associated' OR 'sars-cov-2 variant omicron' OR 'sars-cov-2 variant p.1' OR 'sars-cov-2 variant p.2' OR 'sars-cov-2 variant p.3' OR 'sars-cov-2 variant theta' OR 'sars-cov-2 variant zeta'

Results: 9403

CONCEPT 3:

'breakthrough infection'/exp OR 'reinfection'/exp

Results: 32,787

FINAL: #1 AND #2 AND #3: 135

Web of Science:

CONCEPT 1:

ALL=(SARS-CoV-2 variants)

Results: 3731

CONCEPT 2:

((((((((((((((((((((((((((((((((((((((ALL=(202012-01)) OR ALL=(20A)) OR ALL=(s4n4)) OR ALL=(20B)) OR ALL=(s267f)) OR ALL=(20E)) OR ALL=(20h 5083 v2)) OR ALL=(6111)) OR ALL=(A.1.177)) OR ALL=(A.23.1)) OR ALL=(alpha)) OR ALL=(B.1.1.7)) OR ALL=(B.1.351)) OR ALL=(B.1.427)) OR ALL=(B.1.429)) OR ALL=(B.1.525)) OR ALL=(B.1.526)) OR ALL=(B.1.617.1)) OR ALL=(B.1.617.2)) OR ALL=(beta)) OR ALL=(C.37)) OR ALL=(CAL.20C)) OR ALL=(cluster 5)) OR ALL=(d61g)) OR ALL=(delta)) OR ALL=(e434k)) OR ALL=(epsilon)) OR ALL=(gamma)) OR ALL=(iota)) OR ALL=(kappa)) OR ALL=(l457r)) OR ALL=(lambda)) OR

ALL=(mink-associated)) OR ALL=(omicron)) OR ALL=(P.1)) OR ALL=(P.2)) OR ALL=(P.3)) OR ALL=(theta)) OR ALL=(zeta)

Results: 5, 805, 246

CONCEPT 3:

(ALL=(breakthrough infections)) OR ALL=(reinfection)

Results: 14,268

FINAL: #1 AND #2 AND #3: 151

ProQuest:

CONCEPT 1:

(SARS-CoV-2 variants) AND PEER (yes)
8,496

CONCEPT 2:

((202012-01 OR 20A OR S484K OR 20B OR S256C OR 20E OR (20h 501y v2) OR 614G OR A.1.177 OR A.23.1) AND PEER(yes)) OR ((alpha OR B.1.1.7 OR B.1.351 OR B.1.427 OR B.1.429 OR B.1.525 OR B.1.526 OR B.1.617.1 OR B.1.617.2 OR beta) AND PEER(yes)) OR ((C.37 OR CAL.20C OR (cluster 5) OR D614G OR delta OR E484K OR epsilon OR gamma OR iota OR kappa) AND PEER(yes)) OR ((L452R OR lambda OR mink-associated OR omicron OR P.1 OR P.2 OR P.3 OR theta OR zeta) AND PEER(yes))
4,789,351

CONCEPT 3:

(breakthrough OR (breakthrough infections) OR reinfection) AND PEER(yes)
235,069

FINAL: #1 AND #2 AND #3: 422
